# How to create innovation by building the translation bridge from basic research into medicinal drugs: an industrial perspective

**DOI:** 10.1186/1479-7364-7-5

**Published:** 2013-03-05

**Authors:** Paul G Germann, Alexander Schuhmacher, Juan Harrison, Ronald Law, Kevin Haug, Gordon Wong

**Affiliations:** 1 New Frontier Science, Takeda GmbH, Constance, 78462, Germany; 2 University of Reutlingen, Reutlingen, 72760, Germany; 3 New Frontier Science, Takeda Pharmaceuticals, Palo Alto, CA, 94301, USA; 4 New Frontier Science, Takeda Pharmaceuticals, Chicago, IL, 60601, USA; 5 New Frontier Science, Takeda Pharmaceuticals, Boston, MA, 02467, USA

**Keywords:** Healthcare industry, Corporate venture capital, Open innovation, New frontier science, Translational development, Technology platforms

## Abstract

The global healthcare industry is undergoing substantial changes and adaptations to the constant decline of approved new medical entities. This decrease in internal research productivity is resulting in a major decline of patent-protected sales (patent cliff) of most of the pharmaceutical companies. Three major global adaptive trends as driving forces to cope with these challenges are evident: cut backs of internal research and development jobs in the western hemisphere (Europe and USA), following the market growth potential of Asia by building up internal or external research and development capabilities there and finally, ‘early innovation hunting’ with an increased focus on identifying and investing in very early innovation sources within academia and small start-up companies. Early innovation hunting can be done by different approaches: increased corporate funding, establishment of translational institutions to bridge innovation, increasing sponsored collaborations and formation of technology hunting groups for capturing very early scientific ideas and concepts. This emerging trend towards early innovation hunting demands special adaptations from both the pharmaceutical industry and basic researchers in academia to bridge the translation into new medicines which deliver innovative medicines that matters to the patient. This opinion article describes the different modalities of cross-fertilisation between basic university or publicly funded institutional research and the applied research and development activities within the pharmaceutical industry. Two key factors in this important translational bridge can be identified: preparation of both partnering organisations to open up for new and sometime disruptive ideas and creation of truly trust-based relationships between the different groups allowing long-term scientific collaborations while acknowledging that value-creating differences are an essential factor for successful collaboration building.

## Introduction

**Figure 1:**
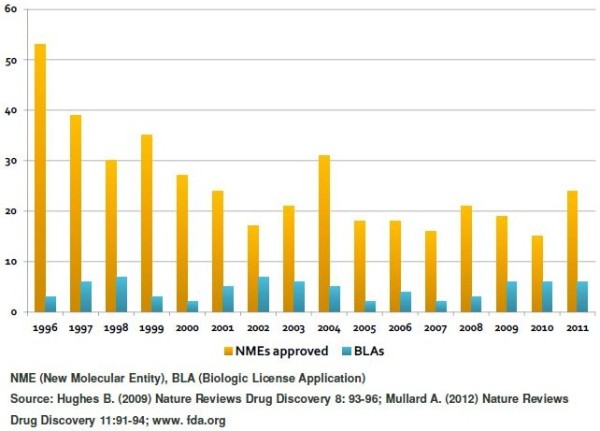
FDA drug approvals.

The human population is facing substantial challenges in the next coming decades with respect to their healthcare: We see an increasing size of the ageing populations, especially in the industrial countries like North America, Europe and Japan. On the other side, there is a clear ‘globalisation’ of healthcare accompanied by an increasing need for improved medication in developing countries. In addition, the constantly increasing costs for the healthcare system leads to a focus towards ‘precise’ medicines. These medicines must have a better therapeutic window, should be personalised to patients needs and accompanied by a validated biomarker concept and might even be combined with other medicines. Therefore, the scope of new research activities in the healthcare system is even broadened by the need to address potentially unfamiliar and complex diseases, new modes of action and challenges like smaller but more distinct multiple patient populations. New therapies will combine pharmaceuticals, diagnostics and devices, with an increase reporting and monitoring of effects via an enhanced IT environment. An increase in innovation is one answer to the upcoming challenges just mentioned. Here, it is important to note that the majority of innovation occurs outside of the pharmaceutical industry. With these upcoming challenges in mind, the global healthcare industry is undergoing substantial changes and adaptations to the constant decline of approved new medical entities (see Figure 1) [1-3].

This decrease in the internal research productivity within most of the larger pharmaceutical companies will prospectively result in a major decline of patent-protected sales (so called ‘patent cliff’) of most of the pharmaceutical companies.

Three major global adaptive trends as driving forces are evident:

1. Cut backs of internal research and development jobs in the western hemisphere (Europe and USA, see Table 1),

2. Following the market growth potential of Asia by building up internal or external research and development capabilities there and

3. ‘Early innovation hunting’ with an opening of the pharmaceutical companies towards very early innovation sources within academia and small start-up companies. This can be done by different means: increased corporate funding, translational institutions to bridge innovation, increasing sponsored collaborations and technology hunting groups for front leading very early scientific ideas and concepts.

**Table 1:** Cut backs of internal research and development jobs in the western hemisphere (Europe and USA), September 2012

**Recent layoffs in big pharmaceutical companies**			
**Ranking by 2011 sales**	**Company**	**Date**	**Notes**
1	Novartis	January 2012	2,000 US sales jobs
2	Sanofi	Imminent	Reportedly up to 2,000 French jobs
3	Pfizer	2005	Still another 12,100 of planned 60,000 jobs to be cut
4	Roche	June 2012	Nutley site to close, 1,000 R and D jobs
5	GlaxoSmithKline	NA	Ongoing restructuring, no specific job target announced
6	Merck & Co	July 2011	12% to13% workforce reduction, in addition to earlier cuts following Schering-Plough takeover
7	Johnson & Johnson	November 2009	7,000 to 8,200 jobs
8	Abbott Laboratories	January 2012	700 manufacturing jobs
9	Bristol-Myers Squibb	NA	Ongoing, 295 jobs cut so far in 2012
10	AstraZeneca	Feb 2012	7,300 jobs, including 2,200 in R and D

*NA* not applicable, *R and D* research and development. Source: Takeda Media Monitoring 19 September 2012.

Especially, this trend of early innovation hunting demands special adaptations not only from pharmaceutical industry, but also from basic research in academia to bridge the translation into new medicines which matters to the patient.

**Figure 2:**
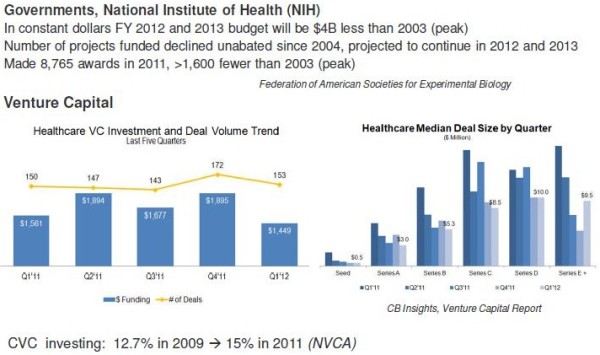
Trends in innovation funding: a US perspective.

On the other side of this innovation bridge, the universities and research institutions will see a static or even a decline in governmental funding, while corporate venture funding takes an increasing stake of importance in the financial system of these research institutions. Corporations are increasingly investing into healthcare as shown in Figure 2.

## Main text

This opinion article describes the different modalities of cross-fertilisation between basic university or publicly funded research institutions and the applied research and development activities within the pharmaceutical industry. Some key phases in this important translational bridge can be identified:

1. *Prepare* the organisations on both sides for opening up for new ideas

2. *Find* the opportunities and *open the doors* to innovation

3. *Create trust* within the scientific collaboration

4. *Develop and select* the winning project as a team

5. *Translate the innovation* into the value chain of a big pharmaceutical company

### Prepare the organisations

The need for a substantial increase of innovative ideas in the health care sector has led to a higher awareness and a closer collaboration between academic institutions and the pharmaceutical industry. The universities/institutions with their technology transfer offices (TTO) are working here as change agents in two directions: by exposing the academics towards external interested partners via ‘open research days’ and business development conferences and by using their manifold networks outside the universities; a mindset change towards a customer orientation is the ultimate goal. In the external direction, fostering of their relationships to newly interested partners is an attempt to seek attention of potential new collaborations and ultimately, funding and partners for the research assets. A challenging point here is the current focus of many TTO and pharmaceutical companies on developed projects with the aim to generate on a fast track mode intellectual property (IP) and a licence option to be granted. Very embryonic assets are normally not in the focus of these kinds of TTO activities.

The hunting and scouting groups of pharmaceutical companies have the critical task to prepare the internal organisation for additional projects to supplement a much-focused high project load of the internal discovery, development and project management organisation. An environment in which external ideas are viewed as threatening competitors to internal project resources needs to be changed by constant communication flow into an open-minded welcoming spirit, which sees the new ideas coming from external as a critical success factor for the whole company. The term ‘landing path’ pictures well this attempt to prepare the internal pharmaceutical company organisation as an open, embracing and welcoming path towards external innovation.

### Find the opportunities and open the doors

Innovation is available in all academic institutions all over the world. Comparing the total sizes of organisations and the total budget spent, academic institutions are outpacing the capacities of the pharmaceutical industry by far. Finding the ‘right’ innovation opportunities, which suits the company’s needs, should use strategic competitive intelligence approaches in a smart and efficient way. The term ‘right opportunity’ will be addressed later. A good general overview of the academic landscape with respect to grant, publication and technology and therapeutic field ranking is the basis. A broad personal network of the company scientists within the selected scientific community is the second important factor to access scientific partners. Thirdly, the building of personal relationships between company and key academic scientists of the selected hot spots of interests are an important factor, which might help to open the right door to the right innovation.

*To create trust within the scientific collaboration* is the key factor of creating a joined success story between academic and pharmaceutical scientists. First of all, in the first introductory meetings, the innovative idea needs to be fully understood by both sides. This means that the colleagues from the pharmaceutical industry have to generate a deep insight into the basic biology or technology to get accepted by the academic collaborator. On the other side, their project and development knowledge and the strategic health benefit perspective, which they bring to the table, might be very beneficial for the academic side as an eye-opener. This kind of relationship building might take several meetings where ongoing discussions might shape more and more the possible joint project. In addition, the pharmaceutical company as a partner can support the academic innovator by giving him/her access to the company compound libraries and connect them to specialised experts and additional technologies. Especially in the case of very early innovative ideas, this phase is a very important process of relationship building as the ‘way forward towards a medicine that matters’ is often not evident and straightforward.

### Develop and select the winning project as a team

During the idea-shaping discussions, the development and translational project knowledge of the pharmaceutical colleagues will be used to generate a project proposal together with the academic inventor. Here three components are essential: in an open and transparent atmosphere, the pharmaceutical company offers support to the academic institution to generate, if not already done, their (academic side!) IP. This should be a unidirectional support from the pharmaceutical company to the academic inventor as a trust-building exercise. This approach of ‘the helping hand’ is not the normal current practice, which might be the reason why a trust building within this early part of a relationship is currently not the easiest way forward. On the other hand, if the project is successful and positive for both sides, then a later licence option already mentioned as the first right of refusal will generate a joined vision for success sharing on both sides.

**Figure 3:**
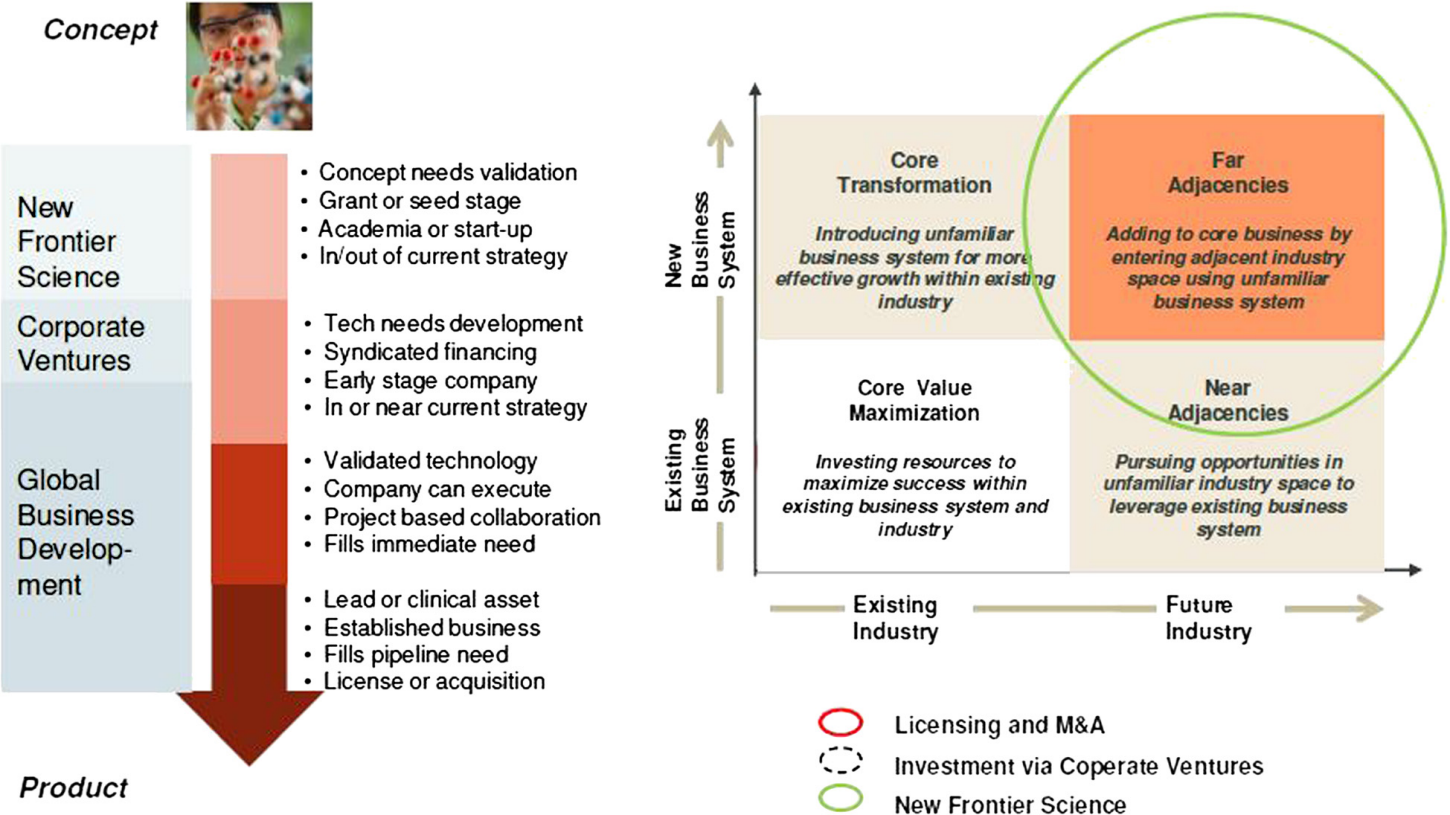
Important deliverables and dependencies in innovation management.

Secondly, in the following discussions, the scope, duration and risk profile of a possible collaboration should be developed. This exercise creates the joint project proposal which defines the collaboration by framing, working tasks, milestones and risks benefits proposal. Within this phase also includes the future phases, in case of collaboration success as well as failure scenarios, which should be transparently addressed. The basic different components for this building relationship are depicted in Figure 3.

If this process is successful, the academic innovator should go out of this phase with the spirit of ‘feeling well supported’, ‘having a partner’ and ‘seeing further than before’. The pharmaceutical partner see the spirit as ‘we are a partner in this project’, ‘let us work on this innovation opportunity’.

Thereby, an important aspect is the ‘buy-in’ process of internal stakeholders in both organisations. Buy-in by both partnering organisations is also a supportive factor for a strong relationship building process.

### Translate innovation into the value chain

To find the right opportunity is an easy sentence with a difficult translation. From the pharmaceutical perspective of pharmaceutical companies, the aim to find the right opportunity and to open the right door is dependent on the corporate strategy. A strong focus on the existing strategic (small molecular entities, also called smols, biologics, therapeutics and diagnostics) and/or therapeutic areas will automatically lead to narrowing the window of innovation hunting. The opportunities found in these more narrow windows are easier to find, select, develop and translate into the own organisation of the pharmaceutical company because of the closeness of the idea towards the receiving organisation and the scientific expertise. Compared to this, innovation outside the ‘comfort zone’ bears a higher risk for scientific error and emotional (not invented here, NIH syndrome) or resource challenges to get acceptance within the organisation. After a certain amount of time exposed to a certain company culture and a constantly high work load, focusing on the own area of project interests can lead to a ‘not invented here’ syndrome. This may lead to a negative bias against innovation coming from the outside of the company zone into its own company.

**Figure 4:**
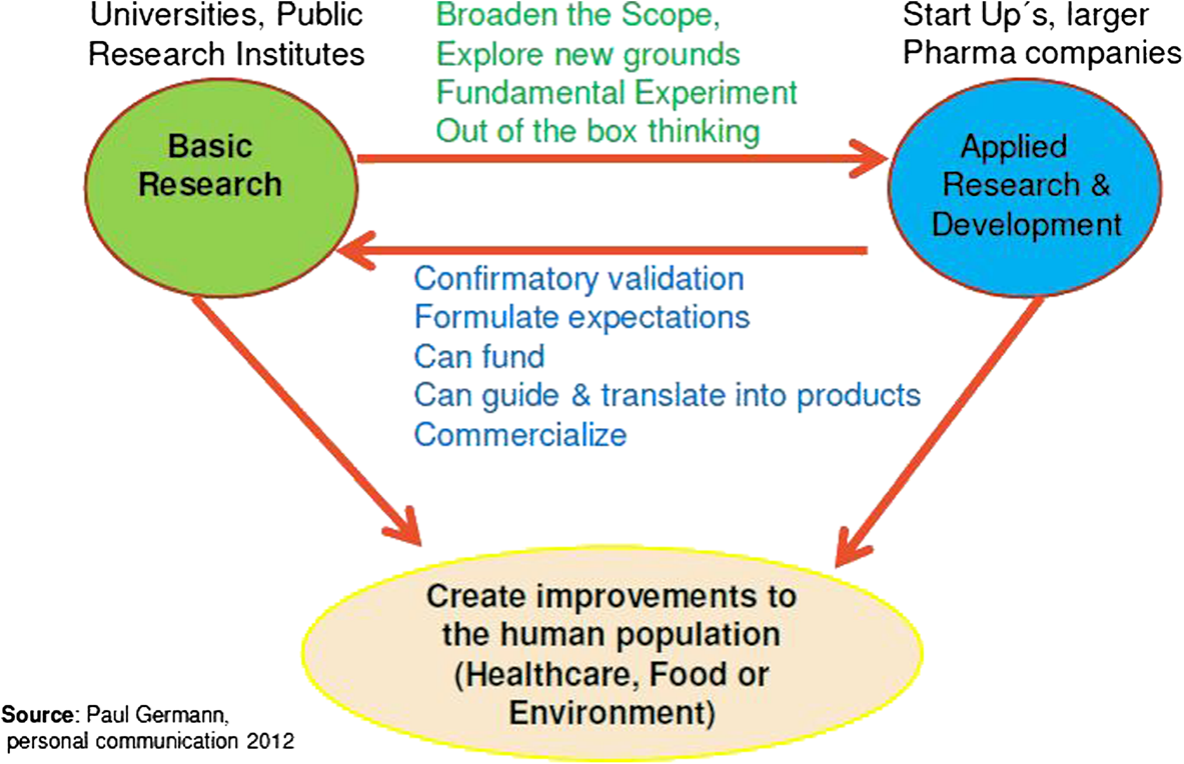
Strategic elements in innovation management.

In addition, early innovative ideas which might need 5 to 15 years to come into product reality need a long breath and a constant internal ‘branding and advertising’ before they might get accepted within the receiving organisation. For example, innovation hunters within the Takeda organisation should work on innovation, which are ‘far adjacencies’ (see Figure 4; PG, personal communication, 2012) to fulfil the strategic focus of the New Frontier Science approach. These innovation hunters should have a special social and communication skills set as depicted in Table 2. To build this critical bridge, social skills like networking building capability are essentially important.

**Table 2:** Soft skill set of innovation hunters


• Active search for innovative solutions to disease	
• Articulate the disease or therapeutic area challenge	
• Radars a range of innovative technologies and solutions	
• Bridges a range risk and time spans to proof of concept	
• Looking for engagement with scientists and inventors	
• Seek engagement with drug discovery units and therapeutic areas	
• Be critical	

Source: Gordon Wong, personal communication 2012.

## Conclusions

The healthcare industry needs a boost of innovative ideas coming from the academic institutions to overcome the current challenges. New ways of collaborations and closer connectivity between the basic research in academic and the applied research within the pharmaceutical industry are essential for a joint success. To build in this broader context, a respectful and trustful relationship between the partners is the key for a win-win situation between academic institutions and pharmaceutical companies.

## Competing interests

PG, JH, RL, KH and GW are employees of Takeda Pharmaceuticals. Therefore these authors, co-authors, would like to declare a conflict of interest in the description of the structural elements of the New Frontier Science concept and strategic elements. AS, employed by the government of the German federal state Baden-Württemberg, Germany, declares no conflict of interest.

## Authors’ contributions

PG drafted the first version of this commentary; the other authors provided editing feedback and main additional data sources. All authors read and approved the final manuscript.

## Authors’ information

PG is an exempt professor at the University for Veterinary Science, Hannover Foundation and currently working as the vice president for New Frontiers Science Europe for Takeda GmbH, Germany.
